# The Osteogenesis of Bone Marrow Stem Cells on mPEG-PCL-mPEG/Hydroxyapatite Composite Scaffold via Solid Freeform Fabrication

**DOI:** 10.1155/2014/321549

**Published:** 2014-04-29

**Authors:** Han-Tsung Liao, Yo-Yu Chen, Yu-Ting Lai, Ming-Fa Hsieh, Cho-Pei Jiang

**Affiliations:** ^1^Department of Plastic and Reconstructive Surgery, Craniofacial Research Center, Chang Gung Memorial Hospital, College of Medicine, Chang Gung University, Kweishan, Taoyuan 333, Taiwan; ^2^Department of Chemical and Materials Engineering, Chang Gung University, Kweishan, Taoyuan 333, Taiwan; ^3^Institute of Mechanical and Electro-Mechanical Engineering, National Formosa University, Yunlin County 632, Taiwan; ^4^Department of Biomedical Engineering, Chung Yuan Christian University, 200 Chung Pei Road, Chung Li 32023, Taiwan; ^5^Department of Power Mechanical Engineering, National Formosa University, Yunlin County 632, Taiwan

## Abstract

The study described a novel bone tissue scaffold fabricated by computer-aided, air pressure-aided deposition system to control the macro- and microstructure precisely. The porcine bone marrow stem cells (PBMSCs) seeded on either mPEG-PCL-mPEG (PCL) or mPEG-PCL-mPEG/hydroxyapatite (PCL/HA) composite scaffold were cultured under osteogenic medium to test the ability of osteogenesis *in vitro*. The experimental outcomes indicated that both scaffolds possessed adequate pore size, porosity, and hydrophilicity for the attachment and proliferation of PBMSCs and the PBMSCs expressed upregulated genes of osteogensis and angiogenesis in similar manner on both scaffolds. The major differences between these two types of the scaffolds were the addition of HA leading to higher hardness of PCL/HA scaffold, cell proliferation, and VEGF gene expression in PCL/HA scaffold. However, the *in vivo* bone forming efficacy between PBMSCs seeded PCL and PCL/HA scaffold was different from the *in vitro* results. The outcome indicated that the PCL/HA scaffold which had bone-mimetic environment due to the addition of HA resulted in better bone regeneration and mechanical strength than those of PCL scaffold. Therefore, providing a bone-mimetic scaffold is another crucial factor for bone tissue engineering in addition to the biocompatibility, 3D architecture with high porosity, and interpored connection.

## 1. Introduction

Bone tissue engineering (BTE) is now a popular research issue for managing the bone defect after tumor ablation, traumatic injury, or osteomyelitis. Basically, the concept of BTE comprises three major parts: scaffold, cell, and signal molecule. Among them, scaffolds play an important role in providing support and space for cell proliferation and differentiation and guiding the surrounding tissue to grow into. Generally speaking, a suitable scaffold for BTE should be a biodegradable, biocompatible, and three-dimensional architecture with porous structure and interpored connection and pose good mechanical strength to support the load-bearing bone [1].

Traditionally, the scaffold was fabricated by phase separation, solvent casting/particulate leaching, fiber meshes, melt-molding/particulate leaching, and gas foaming [2]. The disadvantages are poor control of pore shape, size, porosity and internal geometry, and spatial distribution. Although the combination of conventional method with indirect molds, which were fabricated by rapid-prototyping (RP) system, was proposed to overcome the mentioned drawbacks, it involved a more complicated fabrication process. In addition, the residual chemical solvent in these combination methods will result in inflammatory or toxic effect when applying to human body.

In contrast, rapid prototyping or solid freeform fabrication (SFF) greatly improves the drawbacks derived from conventional techniques [3]. The merits are precise control of pore size, shape, and internal geometry. Furthermore, by assistance of computer-aided design (CAD), it can fabricate the complex, 3D, and anatomic-shape scaffold in a precise, delicate, and reproducible way for either micro- or macrostructure. Stereolithography (SLA), fused deposition manufacturing (FDM), selective laser sintering (SLS), and three-dimensional printing (3DP) are frequently used rapid prototyping systems [4–6]. All of them have their own disadvantages. SLA is based on a UV laser to initiate polymerization of photopolymerisable liquid polymer material, which resulted in the limited choice of photopolymerisable biomaterials for scaffold creation. The process of FDM is to melt the polymer by heat and pump the filament material through a nozzle directly on to the build platform following a programmed path. The disadvantages are the restriction on the use of thermoplastic materials with good melt viscosity properties. SLS uses the laser energy to fuse the biopolymer powders. The disadvantages are hard-to-remove trapped powders and complex and expensive equipment. Three-dimensional printing (3-DP) produces the scaffolds by ink-jet printing a binder into sequential powder layers. The residual harmful binder is hard to remove completely.

In this study, we have tailor-made an air pressure-aided deposition (APAD) system to fabricate the BTE scaffold [7]. Previously, we have demonstrated the successful scaffold fabrication by APAD with biodegradable mPEG-PCL-mPEG triblock (PCL) copolymers and mPEG-PCL-mPEG/hydroxyapatite (PCL/HA) biocomposite [7]. Both scaffolds were shown to provide good support for osteoblasts' attachment and proliferation. However, the HA-containing scaffold is believed to be more bone-mimetic for osteoprogenitor cells because of the increasing mechanical strength and similar chemical composition as bone tissue [8–10]. Hence, the aim of this study is to further investigate the comprehensive BTE application of these two scaffolds both* in vitro* and* in vivo*. Instead of osteoblasts, we chose porcine bone marrow mesenchymal stem cells (PBMSCs) as cell sources because they are more primitive cells with better capacity of proliferation and differentiation. Bone marrow mesenchymal stem cells have been verified on the ability of multilineage differentiation, especially osteogenic potential [11–13]. PBMSCs were seeded on both scaffolds and cultured under osteogenic medium. The first aim was to determine which scaffold was optimal for osteogenesis of PBMSCs by analyzing proliferative ability, cell-scaffold interaction, and the osteogenic gene expression in* in vitro* study. Because the* in vitro* result does not always represent* in vivo* outcome owing to the complicated* in vivo* environment, the second aim was to further compare the bone regeneration capacity between PCL/PBMSCs and PCL/HA/PBMSCs tissue-engineered construct in a pig temporal bone defect model.

## 2. Materials and Methods

### 2.1. Preparation of mPEG-PCL-mPEG Polymer and mPEG-PCL-mPEG Biocomposite/Hydroxyapatite

The mPEG-PCL-mPEG triblock polymer was synthesized as in previous report [7]. In brief, the diblock copolymers were synthesized by ring-opening polymerization of *ε*-caprolactone in the presence of mPEG-OH as a macroinitiator with Sn(Oct)_2_ serving as a catalyst. A predetermined amount of mPEG-OH, *ε*-caprolactone, and Sn(Oct)_2_ was introduced into a 250 mL three-necked flask under a nitrogen atmosphere and mechanical stirring. The resulting mixture reacted for 12 h at 130°C. After the reaction was completed, the mixture was dissolved in dichloromethane (DCM) and precipitated in diethyl ether : hexane (7 : 3, v/v). The product was filtered and dried in a vacuum for 24 h to give mPEG-PCL-OH diblock copolymer first. To synthesize triblock copolymers, the hydroxyl group of mPEG-PCL was modified to a carboxylic acid group. The mPEG-PCL (1 mmol) was dissolved in dry 1,4-dioxane and then an excess of succinic anhydride (2 mmol), TEA (2 mmol), and DMAP (2 mmol) were added to the solution.

The resulting solution was stirred at room temperature for 24 h. The solution was precipitated in diethyl ether : hexane (7 : 3, v/v). The white powder was dried in a vacuum for 24 h. The obtained dried white powder was then dissolved in DCM and stirred at room temperature for 30 min. Then DCC and DMAP were added sequentially. The mPEG-PCL-COOH was dissolved in DCM and introduced to the reaction mixture. The resulting mixture was stirred for 24 h at room temperature under a nitrogen atmosphere. The product was obtained by precipitation in a mixture of diethyl ether and hexane (7 : 3, v/v). The powder was dialyzed against water to remove unreacted residual mPEG and the final product, a white solid powder, was obtained by lyophilization.

For preparation of mPEG-PCL-mPEG/hydroxyapatite biocomposite, the milled HA powder with average size of 100 *μ*m was blended with mPEG-PCL-mPEG polymer by a weight ratio of 0.5.

### 2.2. Air Pressure-Aided Deposition System for Scaffold Fabrication

A desktop air pressure-aided deposition system was used for this study as in previous report [7]. The hardware component consists of an XYZ position system, a material heating module, a temperature control system, and an air pressure control system with an air compressor machine. The system control software directs the material deposition according to the tool path to form a layered 3D scaffold. The heating modulus consists of a container, a nozzle, and a heating holder. It delivers the synthesized copolymer in a molten form through the deposition nozzle with an inner diameter of 0.8 mm. The powder form of the synthesized copolymer is then melted, at a temperature of 65°C, higher than the melting temperature of the synthesized copolymer, by heating the holder through the temperature control system. The molten copolymer is finally deposited as a result of the pressure supplied by the air pressure control system. The dimensions of the bone scaffold designed were 20 mm × 20 mm × 10 mm, meaning that its interconnection was also 10 mm long.

The comparison of scaffold properties such as pore size, porosity, mechanical strength, and hydrophilicity was done as in previous report [7]. The PCL/HA scaffold was further analyzed by microcomputer tomography (micro-CT) for the inner pore structure and interpored channel.

### 2.3. Isolation of Porcine Bone Marrow Derived Stem Cells

PBMSCs were harvested and isolated according to the procedure previously described [13–15]. All experiments were approved by Chang Gung Memorial Hospital's Institutional Animal Care and Use Committee and follow the guidelines of experimental animal care. Briefly, we aspirated bone marrow from a pig's iliac crest by using an aspiration syringe filled with heparin (1000 U/mL bone marrow) to prevent clotting. Retrieved bone marrow was plated in cell culture flasks containing the normal growth medium (Dulbecco's modified Eagle medium (DMEM) supplemented with 100 U/mL penicillin, 100 *μ*g/mL streptomycin, and 10% fetal bovine serum (FBS)). Porcine BMSCs attached to the culture flask were passaged when cells were 80% confluent and expanded for 10–12 days with medium change every 3 d.

### 2.4. Comparison of Osteogenesis of PBMSCs among Different Scaffolds

Second passage PBMSCs were used for* in vitro* studies of osteogenic differentiation. To induce osteogenic differentiation, the cells/scaffold constructs were cultured in medium containing osteogenic supplements (DMEM with 10% fetal bovine serum, 100 nmol/L dexamethasone, 50 *μ*g/mL ascorbic acid, and 10 mmol/L-glycerophosphate). A disk-shape PCL and PCL/HA scaffold (5 mm in thickness and 10 mm in diameter) was sterilized by 75% alcohol followed by UV irradiation for one day and washed with sterilized PBS several times to remove residual alcohol before cell seeding. The experiments were divided into two groups. Group I (PCL/PBMSCs): PBMSCs (~1 × 10^5^ cells) were seeded in PCL scaffolds and cultured in osteogenic medium. Group II (PCL/HA/PBMSCs): PASCs (~1 × 10^5^ cells) were seeded in PCL scaffolds and cultured in osteogenic medium. The cell proliferation, Live/Dead test, osteogenic mRNA expression of Runx-II, osteocalcin (OCN), ALP, and angiogenic mRNA expression (VEGF) were compared between two groups at 0, 7, 14, and 21 days. Day 0 represented 6 h after cell seeding on the scaffold.

#### 2.4.1. Cell Proliferation

The CellTiter 96 AQueous One Solution Reagent (Promega Co., Madison, WI, USA), which contains a novel tetrazolium salt (MTS), was used to determine the proliferation of PBMSCs at each group. The MTS tetrazolium compound is reduced by living cells into a colored formazan product that is soluble in tissue culture medium. The quantity of formazan product is directly proportional to the number of viable cells. MTS assays were performed by adding 20 *μ*L of MTS solution to each specimen in 100 *μ*L culture medium and incubating at room temperature for 3 h with protection from light. Colorimetric measurement of the formazan dye was performed at a wavelength of 492 nm using an ELISA plate reader (Molecular Devices, Sunnyvale, CA, USA). Cell numbers were determined using a calibration curve relating the number of PBMSCs and the absorbance value.

#### 2.4.2. Cell Viability Test

The viability of PBMSCs in each group was assessed by the Live/Dead Viability/Cytotoxicity Assay Kit. The Kit provides two molecular probes, calcein AM and ethidium homodimer-1 (EthD-1), to simultaneously determine the existence of the live and dead cells, based on the intracellular esterase activity and plasma membrane integrity. Live cells emitted green fluorescent light because those cells were permeated with nonfluorescent calcein AM and followed by intracellular hydrolysis by esterase to give fluorescent calcein. EthD-1 enters cells with damaged membrane and binds to nucleic acids to produce a bright red fluorescence in dead cells. The fluorescence-stained cells were imaged using an Inverted Fluorescence Microscope (Leica DMIL). The excitation wave-lengths for calcein AM and EthD-1 are 494 and 528 nm, respectively. The emission wavelengths for calcein AM and EthD-1 are 517 and 617 nm, respectively. The viability of PBMSCs was compared between both groups at time point of 0, 7, 14, and 21 days.

#### 2.4.3. Real Time PCR of mRNA Expression of Osteogenic Protein

Total RNA of each specimen was isolated with Trizol reagent (Invitrogen, Carlsbad, CA, USA) following the manufacturer's protocols. Isolated RNA was dissolved in RNase-free water and the amount of RNA was determined by measuring the absorbance value (OD) at 260 nm with a spectrophotometer. RNA quality was verified by measurement of OD260/OD280. The cDNA was prepared from 2 *μ*g of total RNA with RevertAid First Strand cDNA Synthesis Kit (Thermo Scientific Molecular Biology) in a final volume of 20 *μ*L. Specific osteogenic marker genes, Runx II, ALP, and osteocalcin (OCN) were tested for osteogenesis. VEGF gene was used for the angiogenic factor expression. The primers sequence for RunX II (forward primer: 5′-GAGGAACCGTTTCAGCTTACTG-3′ and reverse primer: 3′-CGTTAACCAATGGCACGAG-5′); ALP (forward primer: 5′-ATGAGCTCAACCGGAACAA-3′, reverse primer: 3′-GTGCCCATGGTCAATCCT-5′) and OCN (forward primer: 5′-TCAACCCCGACTGCGACGAG-3′; reverse primer: 3′-TTGGAGCAGCTGGGATGATGG-5′); VEGF (forward primer: 5′-CTCTACCTCCACCATGCCAAG and Reverse primer: 3′-AGACATCCATGAACTTCACCACTTC-5′); GAPDH (forward primer: 5′-GCTTTGCCCCGCGATCTAATGTTC-3′ and reverse primer: 3′-GCCAAATCCGTTCACTCCGACCTT-5′) were designed using the Oligo 6.0 program (Molecular Biology Insights, Inc., Cascade, CO, USA). For a single PCR reaction amounting to 20 *μ*L, 0.2 *μ*L of cDNA was used. To make the visualization of PCR products possible in real time, a SYBR Green I supermix (Yeastern Biotech Co., Taipei, Taiwan) was used. A three-temperature cycling, consisting of a denaturation step at 95°C for 30 s and annealing step at 57.6°C for 30 s and extension step at 72°C for 30 s, was carried out in an iCycler iQ5 real-time detection system (Bio-Rad Laboratories Inc., Hercules, CA, USA). The specificity of each PCR reaction was assessed by performing melting curve analysis after each reaction. Glyceraldehyde-3-phosphate dehydrogenase (GAPDH) acted as a housekeeping control. Results were quantified for marker genes using the 2 − ΔΔ*Ct* relative quantification method with respect to the control time point at day 0. The expression of each gene was evaluated in triplicate.

#### 2.4.4. Cell-Scaffold Interaction Observation by SEM

The interaction between adhered PBMSCs at each group was examined by SEM analysis. Culture medium was removed completely by washing with sterile PBS. Furthermore, 2.5% glutaraldehyde solution was added in each well for 16 h at room temperature to fix the adhered cells onto the scaffold surface. After mild washing with PBS, the scaffold sections were dehydrated by gradually increasing concentration of ethanol (20%, 40%, 60%, 80%, and absolute ethanol) for 10–15 min each. The dehydrated scaffold sections were placed in vacuum desiccators for complete drying. Surface of cell-adhered scaffold was observed by SEM analysis after coating with gold using sputter coater.

### 2.5. *In Vivo* Animal Study

**Figure 1:**
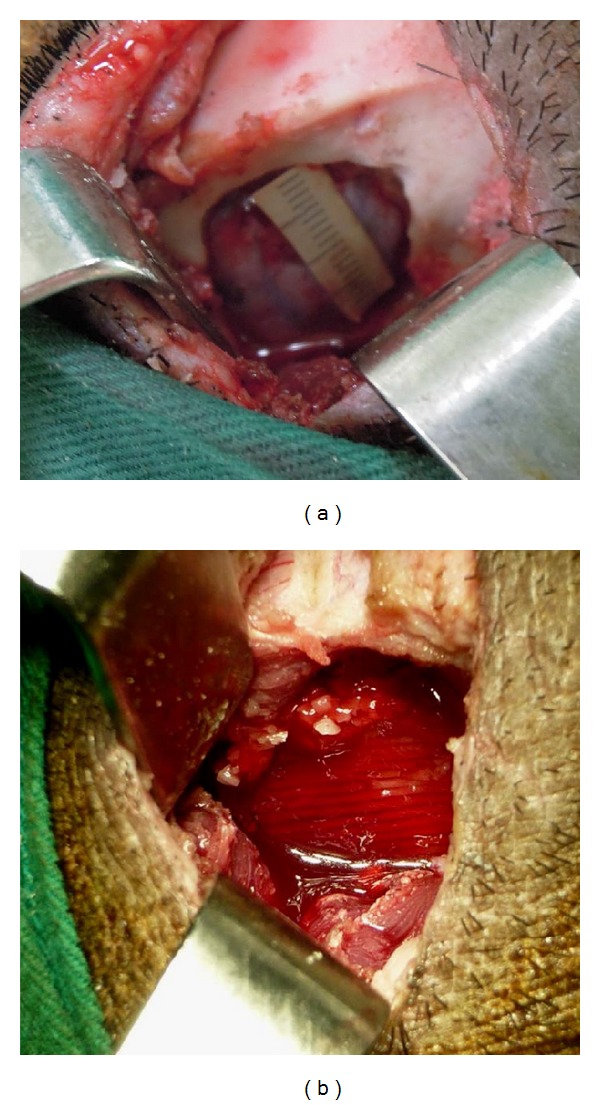
The surgery procedure for bone regeneration with a pig temporal bone defect model. (a) A full-thickness 2 cm × 2 cm bone defect was created at temporal bone area of a pig, which was reconstructed by (b) a tissue-engineered PCL/HA/PBMSCs construct.

All animal experiments were approved by Chang Gung Memorial Hospital's Institutional Animal Care and Use Committee and followed the guidelines of experimental animal care. Surgery was performed under sterile conditions by using endotracheal isoflurane anesthesia after induction with intravenous 4% sodium pentobarbital (1 mL/10 kg). The donor area of the scalp in each animal was shaved before the procedure, and the surgical field was prepared with betadine solution. The bilateral temporal fossae were exposed through a coronal incision. A full-thickness square bone defect, 2 × 2 cm, was created bilaterally at temporal bone by using a bur under extensive cooling with saline (Figure 1(a)). The defects were divided into two groups as follows. Group I: the defect was implanted with PCL scaffold and 1 × 10^8^ PBMSCs. Group II: the defect was implanted with PCL/HA scaffold and 1 × 10^8^ PBMSCs (Figure 1(b)). After achieving homeostasis, the wound was resutured with 3-0 dexon and 3-0 nylon by layered closure. Cefazolin (100 mg/kg) was administered preoperatively, immediately after surgery, and 24 hours later. To alleviate postoperative pain, the animals were given Buprenex (0.01 mg/kg, intramuscularly) every 12 hours over 48 hours. The wound was cleared and treated with neomycin ointment during the healing period.

The animals were sacrificed 6 months after implantation under anesthesia with overdosed pentobarbital and the implants were harvested. Three-dimensional computer tomography (3D-CT) was used to evaluate the bone regeneration in the defect 1 week and 2, 4, and 6 months after operation. The Vickers diamond microhardness test was used to measure the stiffness of new regenerated bone at each group. Histological stain by H&E and Masson's trichrome stain were done to confirm if the regeneration tissue was bone tissue.

#### 2.5.1. Computerized Tomography Analysis

All animals underwent postoperative computerized tomography (CT) examination at 1 week and 2, 4, and 6 months after the operation. Computed tomography parameters included a slice thickness of 1.25 mm with a 0.625 mm interval, a tube voltage of 120 kVp, and a tube current of 300 mA. The CT image acquisition, processing, and manipulation were performed according to the standard protocol at this medical facility. The CT data were reformatted, and a voxel (unit of three-dimensional (3D) image) was set at 0.6 × 0.6 × 0.6 mm for all scans. The imaging data were analyzed using the Osirix Image software (version 3.6.1) for comparing the new bone regeneration in 2D cross-section and 3D view among groups. The craniofacial bone was extracted from the 3D CT images with the threshold adjusted to remove the soft tissue and display the bone. The range of CT densities was fixed in all CT scans for craniofacial bone of each animal.

Tissue mineral density was evaluated by Hounsfield unit (HU). Briefly, the Osirix image software was used to calculate the average HU value by outlining the region of interest that was confined to the bone defect region at 2D cross-sectional view. Each group was measured with eight repeats and normal bone surrounding defect was measured as control.

#### 2.5.2. Histological Examination

Samples were decalcified first and then examined by histology for bone formation by fixing in 4% paraformaldehyde, dehydrated, and embedded in paraffin. Four-micrometer-thick serial sections were obtained and subject to H&E and Masson's trichrome stains.

### 2.6. Statistical Analysis

All data are reported as mean ± standard deviation. Statistics among multiple groups on cell proliferations and biochemical assays were carried out using one-way ANOVA test to determine significant differences. Tukey's post hoc test was used to determine the difference between any two groups with *P* < 0.05 considered statistically significant.

## 3. Results

### 3.1. Scaffolds Fabrication and Character

**Figure 2:**
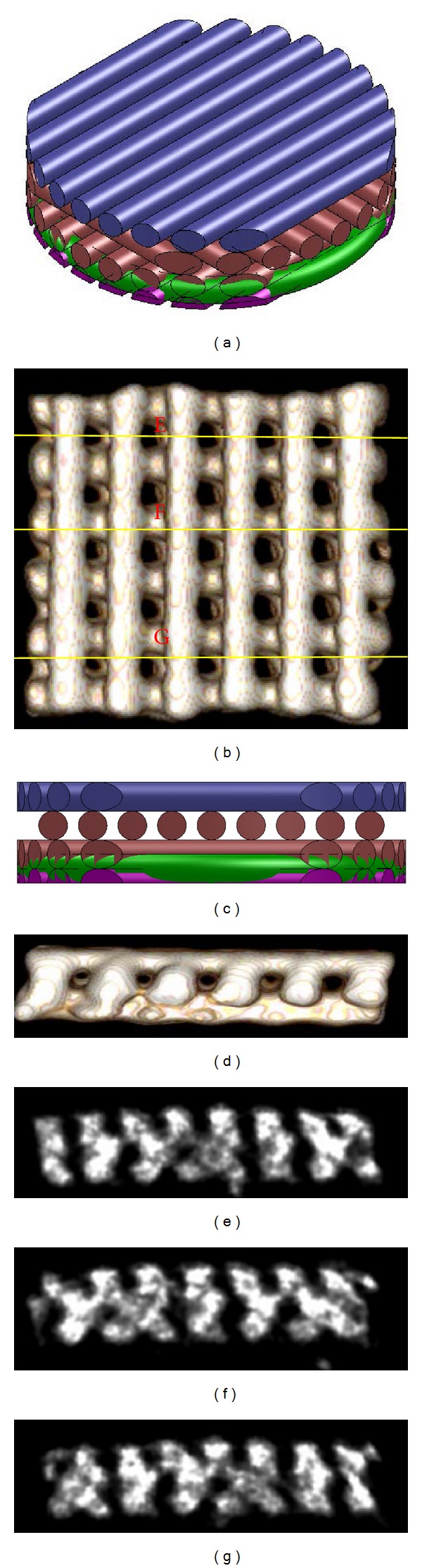
Illustration of scaffold design: (a) frontal view, (c) cross-sectional view; microcomputed tomography (*μ*-CT) analysis of PCL/HA scaffolds prepared by APAD. (b) Frontal view, (d) cross-sectional view. The lines in 3D image (b) mark the planes of cross-sections where 2D cross-sectional views are taken ((e), (f), and (g)).

The frontal and cross-sectional view of scaffold design was illustrated as in Figures 2(a) and 2(c), respectively. The PCL/HA scaffold fabricated by APAD was further analyzed by micro-CT. After 3D reconstruction by Osirix software, Figures 2(b) and 2(d) represent the frontal and cross-sectional view of the PCL/HA scaffold. These results confirmed the fabricated scaffold by APAD was identical as the illustration (Figures 2(a) and 2(c)). For realization of the internal pore structure and interporous connection, the 2D cross-sectional view was observed at upper, middle, and lower level of scaffold by Osirix software. All the three levels showed the regular pore structure with interpored connection (Figures 2(e), 2(f), and 2(g)).

The character of PCL and PCL/HA scaffold was similar to our previous report [7]. The pore size of PCL was 327.32 ± 5.46 *μ*m and the PCL/HA was 374.32 ± 11.25 *μ*m. The porosity of PCL and PCL/HA scaffold was 79 and 80%, respectively. The contact angle was 84.5° and 88.2° at PCL and PCL/HA scaffold, respectively. The compressive strength was 9.28 and 18.38 Mpa at PCL and PCL/HA scaffold, respectively.

### 3.2. *In Vitro *Experiments

#### 3.2.1. MTS Assay and Cell Viability

**Figure 3:**
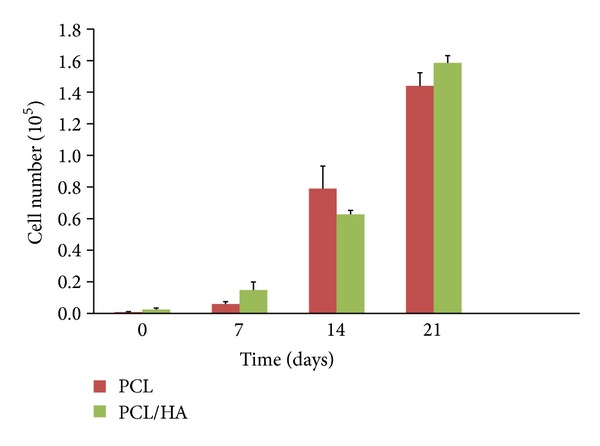
Comparison of cell proliferation of porcine bone marrow stem cells (PBMSCs) in PCL and PCL/HA scaffolds at different time points.

**Figure 4:**
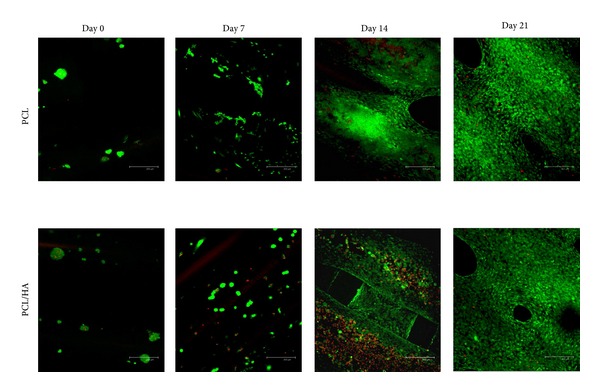
The viability of PBMSCs at PCL and PCL/HA group from day 0 to day 21 (green means live cells and red means dead cells); scale bar: 100 *μ*m.

The results of the MTS assay (Figure 3) showed that the numbers of PBMSCs gradually increased from day 0 to day 21 at both groups. The PBMSCs had significant optimal proliferation (*P* < 0.05) at day 21 compared to days 14, 7, and 0 and the proliferation at day 14 was also significantly higher than day 7 and day 0 at both groups. No significant difference was found between days 0 and 7 at both groups. For each culture period, significantly higher proliferation of PCL/HA than that of PCL was found at days 14 and 21. The Live/Dead assay (Figure 4) also showed comparable results as the MTS assay did. The viable cells (green color) gradually increased from day 0 to day 21 at both groups with only few dead cells (red color).

#### 3.2.2. Real Time PCR of Osteogenic and Angiogenic mRNA

**Figure 5:**
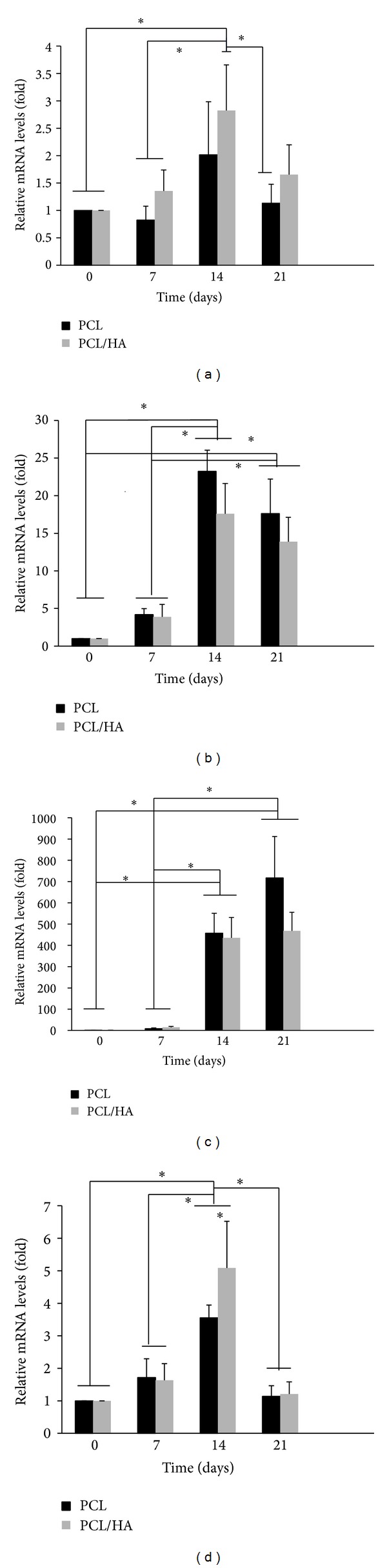
Relative (a) Runx II, (b) alkaline phosphatase (ALP), (c) osteocalcin (OCN), and (d) VEGF mRNA expression of PBMSCs in PCL and PCL/HA scaffolds at different time points. The relative qRT-PCR values were corrected using the glyceraldehyde-3-phosphate dehydrogenase (GAPDH) expression levels and normalized with respect to the values on day 0 of culture. The values are the mean values ± SD of three independent experiments. *P < 0.05.

The expression of osteogenic mRNA gene (ALP, RunX II, and OCN) in both groups was all upregulated after culturing on osteogenic medium (Figures 5(a), 5(b), and 5(c)). The ALP and RunX II gene reached the maximum expression at day 14. The OCN gene showed a time-dependent upregulation from day 0 to day 21. No significant differences were found between two groups at either any gene or time point. The angiogenic gene expression (VEGF) was upregulated gradually from day 0 to day 14 and declined at day 21 (Figure 5(d)). The PBMSCs demonstrated significantly higher VEGF expression at PCL/HA group than at PCL group at day 14.

#### 3.2.3. Cell-Scaffold Interaction

**Figure 6:**
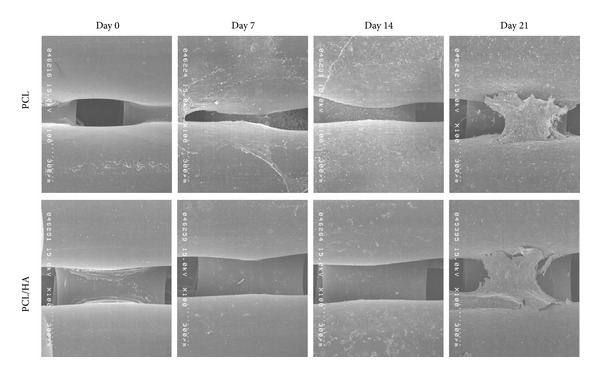
The SEM showed the cell-scaffold interaction at PCL and PCL/HAP group from day 0 to day 21.

SEM was used to observe the attachment and spreading of the PBMSCs at different time points in both groups.  Figure 6 showed that PBMSCs attached and spread well on both scaffolds (Figure 6). At day 21, the PBMSCs grew and spread fully on the backbone of both scaffolds which was identical to the outcome of Live/Dead assay.

### 3.3. *In Vivo* Animal Study

**Figure 7:**
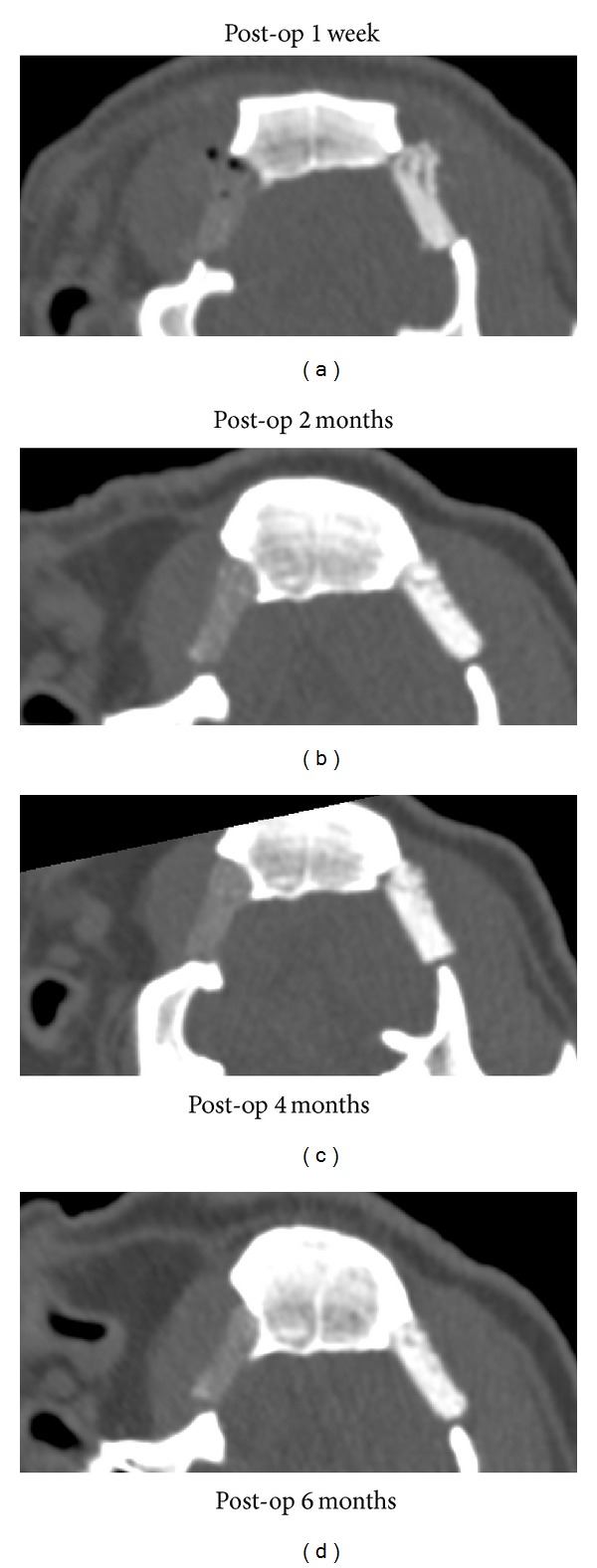
The coronal view of two-dimensional view of bilateral pig bone defects which were reconstructed by PCL/PBMSCs at left side and PCL/HA/PBMSCs at right side. (a) Post-op 1 w, (b) post-op 2 months, (c) post-op 4 months, and (d) post-op 6 months.

**Figure 8:**
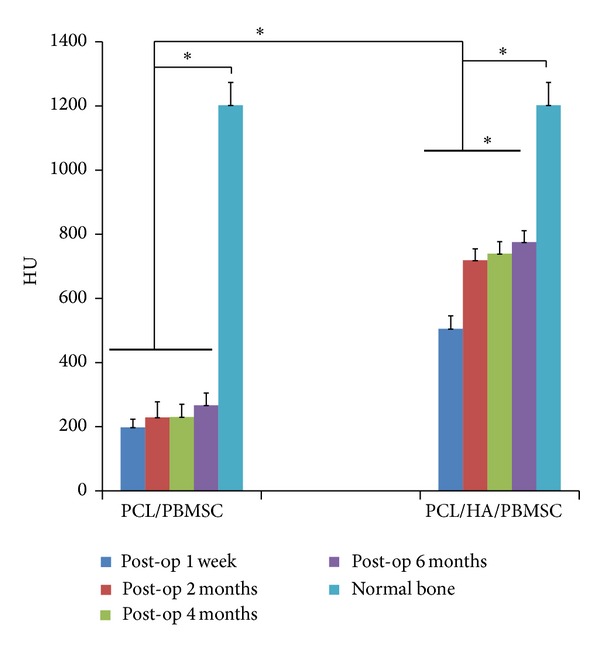
Comparison of Hounsfield units (HU) among PCL/PBMSCs, PCL/HA/PBMSCs groups at post-op 1 w, 2 m, 4 m, and 6 m and normal bone.

The through and through 2 × 2 cm square bone defects on bilateral temporal fossae were reconstructed by PCL/PBMSCs and PCL/HA/PBMSCs at left and right side, respectively. The pigs recovered evenly after operation. No infection, wound dehiscence, implant extrusion, or death was found during follow-up period. The pigs underwent CT for evaluating the bone formation at 1 wk and 2, 4, and 6 mo post-op. The CT images showed less image density in the PCL/PBMSCs than PCL/HA/PBMSCs group at post-op 1 week (Figure 7). It was because the hydroxyapatite will enhance the density of PCL/HA scaffold. However, the density of PCL group was not increased very prominently from 2, 4, and 6 months after operation comparing to 1 week postoperation (Figure 7). Conversely, the image density of PCL/HA groups elevated obviously from 2, 4, and 6 months after operation comparing to 1 week postoperation (Figure 7). For quantification of the mineralization and density at implant area, the Hounsfield units (HU) with 8 repeated data were calculated at both groups and normal bone by Osirix software (Figure 8). The outcome showed the HU were gradually increased with time-dependent manner at both groups. The HU at PCL/HA was significantly higher than PCL group at all time point. However, the time-dependent elevation was not significant at PCL group. Conversely, at PCL/HAP group the increasing at post-op 2, 4, and 6 mo was significantly higher than post-op 1 wk. Comparing to the HU of normal bone, the HU of PCL/PBMSC and PCL/HA/PBMSC group at 6 months was 22% and 64% of that of normal bone, respectively.

**Figure 9:**
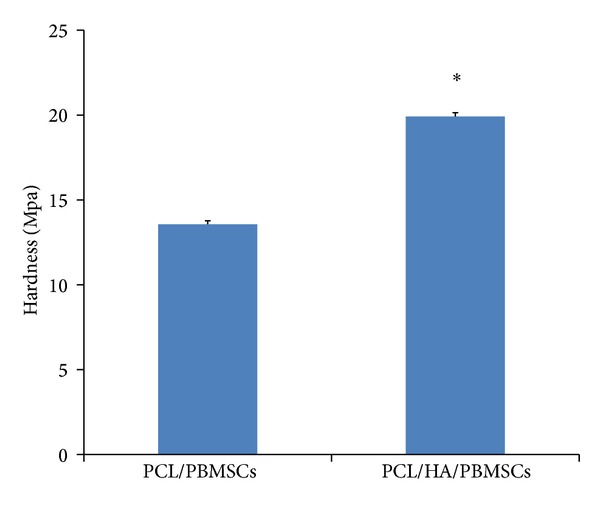
Comparison of the hardness of harvested specimens between PCL/PBMSCs and PCL/HA/PBMSCs after 6-month implantation in pig temporal bone.

**Figure 10:**
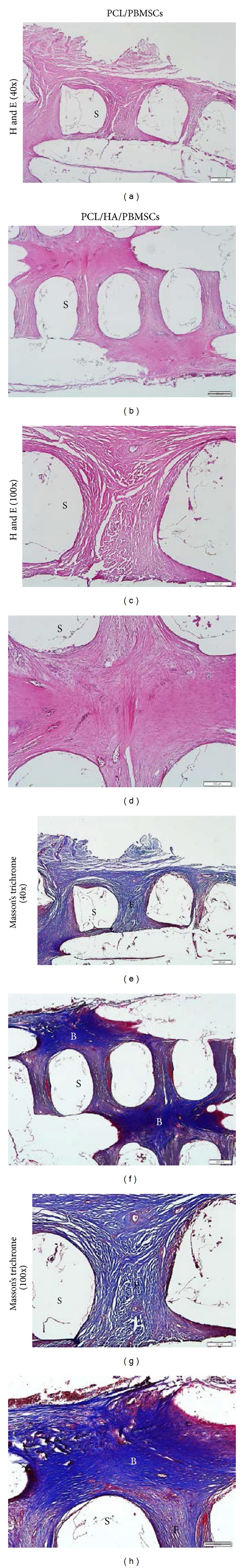
The histological examination of specimens post-op 6 months for PCL/PBMSCs: (a) H&E (40X), (c) H&E 100X, (e) Masson's trichrome stain (40X), and (g) Masson's trichrome stain (100X) and PCL/HA/PBMSCs group: (b) H&E (40X), (d) H&E 100X, (f) Masson's trichrome stain (40X), and (h) Masson's trichrome stain (100X). S: scaffold; B: bone formation area (deep blue area); F: fibrotic tissue area (light blue area). Black scale bar at 40X is 200 *μ*m and black scale bar at 100X is 100 *μ*m.

The pigs were sacrificed at post-op 6 mo and the implant tissue-engineered bone construct was harvested for the mechanical strength and histological analysis. The Vickers diamond microhardness test revealed that the PCL/HA group had significantly higher mechanical strength than PCL group (Figure 9). The histology also showed more bone formation (deep blue area in Masson's trichrome stain) within the pore of scaffold at PCL/HA group (Figures 10(b), 10(d), 10(f), and 10(h)) than PCL group (Figures 10(a), 10(c), 10(e), and 10(g)) by both H&E and Masson's trichrome stains.

## 4. Discussion

Although the optimal scaffold for BTE is not discovered yet, several characters are required including biocompatibility, biodegradability, three-dimensional architecture, high porosity, and interpored connection. In this study, we used our in-built solid free form system (APAD) to fabricate a three-dimensional architecture with pore structure and interpored connection. Because the system is computer-aided, the scaffold can be fabricated precisely in macro- and microstructure repeatedly. Figures 1(b) and 1(d) showed the identical architecture as the illustration. Figures 1(e), 1(f), and 1(g) also addressed the constant pore structure and interpored connection controlled by computer program. These merits prevent the drawbacks derived from traditional fabrication methods including the variable pore size with unpredictable interpored connection and different internal microstructure from batch to batch.

The main materials in our scaffold are PCL, PEG, and HA. PCL is easy to apply for clinical setting in the future because it is approved by FDA and can be degraded by human body without the production of toxic degradants. However, pure PCL is relatively hydrophobic with an average contact angle of 119.2°, which is not suitable for cell attachment [16]. Hence, we modified the PCL by coupling it with mPEG into mPEG-PCL-mPEG triblock copolymer. PEG is also a well-known biocompatible biomaterials and more importantly, it is hydrophilic. With the modifications by hydrophilic PEG, the contact angle was dramatically dropping to 84.6 and 88.2 in mPEG-PCL-mPEG and mPEG-PCL-mPEG/HA scaffold, respectively. These revealed that both scaffolds became more hydrophilic after the modifications. Adding HA in mPEG-PCL-mPEG scaffold only slightly influences the hydrophilicity.

Pore size and porosity of scaffold are other important factors for successful BTE. High porosity with adequate pore size provides adequate void spaces for surrounding bone as well as vascularized tissue to grow into. The minimal pore size for regeneration of mineralized bone tissue was described as at least 100 *μ*m [17]. Oh et al. indicated the influences of the pore size gradient of PCL scaffold from 88 to 405 *μ*m for bone regeneration on rabbit skull defect [18]. The results showed the pore size of 380–405 *μ*m had better cell growth for osteoblasts. The small pores resulted in osteochondral formation first before osteogenesis; conversely, large pores lead to direct osteogenesis. The reasons might be that the small pore size indicated relative hypoxic condition with low oxygen content and large pores resulted in high oxygen content by quickly simultaneously vascularization. Karageorgiou and Kaplan concluded that pore size >300 *μ*m is recommended because of enhancement of new bone and capillaries formation [19]. Roy et al. described a pore size-dependent relationship to new bone formation and gradual elevation of new bone formation was found from 80% to 88% [20]. The pore size of PCL scaffold was 327.32 ± 5.46 *μ*m and the PCL/HA scaffold was 374.32 ± 11.25 *μ*m. The porosity of PCL and PCL/HA scaffold was 79 and 80%, respectively. Taken together, the pore size and porosity of both scaffolds in our study seemed to be suitable for BTE.

Mechanical strength is one of the important determinant factors of scaffold performance for BTE. Although the cranial bone is not a load-bearing area, it still requires adequate stiffness to protect the inner vulnerable brain tissue. The mechanical strength of scaffold is influenced by porosity, internal geometry, and material itself. Yeong et al. found an inverted relationship between porosity and compressive Young's modulus of their porous PCL scaffold [21]. Higher porosity will cause lower mechanical strength. Eshraghi and Das also described that the porosity of PCL scaffold ranging from 51.1% to 80.9% exhibited the compressive strength ranging from 10.0 to 0.6 Mpa [22]. However, high porosity is very important for bone TE to provide space for cell proliferation, vascular ingrowth, and nutrient and waste exchange. Hence, for increasing the mechanical strength without sacrificing the high porosity, we added the hydroxyapatite into the backbone of mPEG-PCL-mPEG scaffold with a weight ratio of 0.5. The mechanical strength showed around 2 times elevation after adding hydroxyapatite to mPEG-PCL-mPEG copolymer. Liao et al. also found the mechanical strength of PCL scaffold fabricated by laser-sintering increased dramatically after adding 30% *β*-tricalcium phosphate [23]. Actually, hydroxyapatite is the major inorganic mineral within bone tissue. Recently, the hydroxyapatite is found to be not only osteoconductive but also osteoinductive [24]. Hence, it makes the scaffold more biomimetic by adding hydroxyapatite into the mPEG-PCL-mPEG.

In our previous report, both mPEG-PCL-mPEG and mPEG-PCL-mPEG/hydroxyapatite scaffolds showed good biocompatibility and proliferation for osteoblasts, but there was slightly better proliferation on mPEG-PCL-mPEG/hydroxyapatite scaffold [7]. In order to verify the ability of both scaffolds in BTE application, we seeded the PBMSCs on both scaffolds and induced them on osteogenic medium for* in vitro* study and implant both scaffold/cells constructs into pig temporal bone defect for* in vivo* study. The cell proliferation of PBMSCs in both groups showed similar outcome as found in our previous osteoblasts experiment. The PBMSCs proliferated gradually from day 0 to day 21 in both groups with higher proliferation in PCL/HA group. The Live/Dead test also confirmed the biocompatibility of both scaffolds with less dead cells on them and gradual spreading and growing of PBMSCs from day 0 to day 21. SEM also showed the compatible result with Live/Dead test. The cells were spreading and growing well on the surface of both scaffolds at day 21. All these evidences indicated both PCL and PCL/HA scaffolds had good biocompatibility for attachment and proliferation of PBMSCs.

The osteogenic differentiation of PBMSCs on both scaffolds was analyzed by the expression of osteogenic specific genes. Runt-related transcription factor 2 (Runx II) is thought to play central role to regulate the stem cells into osteoblastic phenotype. Runx II directly stimulates the expression of downstream osteogenic genes such as osteocalcin, osteopontin, collagen I, bone sialoprotein, and alkaline phosphatase (ALP) [25–27]. Although ALP is not an osteogenic-specific gene, it is always gradually expressed in the early period of osteogenic differentiation and declined in late period [28]. Osteocalcin (OCN) is a bone-specific gene and is one of the most abundant proteins in the bone, secondary only to collagen type I [27]. The OCN is upregulated during matrix synthesis and mineralization. In this study, the osteogenic gene expression of PBMSCs was in a similar pattern on both scaffolds. The Runx II and ALP gene expressed gradual increasing from day 0 to day 14 with the maximum expression at day 14 and then declined. The OCN gene was expressed as a time-dependent manner with gradual increasing from d 0 to d 21. No significant differences were found between two groups at each gene and each time point. It was believed that the Runx II and ALP genes were expressed in early stage and OCN in late stage of osteogenic differentiation; our results also confirmed the temporal change of the gene expression during osteogenic differentiation. In summary, the PBMSCs were successfully induced toward osteogenic differentiation on both scaffolds.

Besides, angiogenesis also plays a crucial role on the bone TE. Usually the BMSCs only can survive on the surface of scaffold due to limited diffusion within 150 to 200 *μ*m from the blood supply source. The speed of fully developed vascularization throughout the whole bone TE scaffold is the key factor for survival of BMSCs especially on the innermost part of scaffold. Angiogenic factors such as VEGF can encourage the ingrowth of the vascular endothelium and further accelerate the establishment of microcirculation within the TE scaffold. Street et al. also described that the inhibition of VEGF resulted in impairment of fracture healing and bone formation [29]. The gradual elevation of VEGF gene expression of PBMSCs from d 0 to d 14 was observed on both scaffolds. The significant higher VEGF gene expression was found in PCL/HA than in PCL group at day 14. This is consistent with the results of He et al.'s report [30]. They also found that the endogenous secretion of vascular endothelial growth factor was sustained at significantly higher levels for human MSCs seeded on HA-contained PLG scaffold than non-HA-containing scaffold. Although the mechanism why hydroxyapatite can enhance the VEGF secretion of BMSCs is still unclear, we assume it is owing to that the hydroxyapatite-contained scaffold produces similar environment as bone tissue, in which 70% is composed by hydroxyapatite. In addition, He et al. also found higher ratio of HA-containing scaffold (2.5 : 1 and 5 : 1 HA : PLG ratios) had a constant and long-term VEGF expression; in contrast, lower ratio composites (0 : 1 and 1 : 1 HA:PLG ratios) showed short-term VEGF expression whose peak was at day 14 [30]. Since the ratio of HA to PCL in our study is 1 : 1, it is the reason why the VEGF gene expression gradually increases with the peak at day 14 and then declines in HA-containing scaffold.

Although the PBMSCs showed similar osteogenesis and angiogenesis pattern on both PCL and PCL/HA scaffolds, the* in vitro* results do not always reflect the tissue response of animal experiments due to the complicated and complex environment within animal body. Rai et al. also found the differences between* in vitro* viability and osteogenic differentiation and* in vivo* bone-forming efficacy of human mesenchymal stem cells cultured on PCL-TCP scaffolds [31]. For this reason, we created a temporal bone defect in large animal (porcine) to compare the bone regeneration capacity between these two groups under the human-like environment. The outcome of CT showed less density in the PCL/PBMSCs than the PCL/HA/PBMSCs group at post-op 1 week. It was because the hydroxyapatite itself has high image density and PCL has low image density in CT scan. However, the density of PCL/PBMSCs group was not increased very prominently from 2, 4, and 6 months after operation comparing to 1-week postoperation. Conversely, the density of PCL/HA/PBMSCs groups elevated obviously from 2, 4, and 6 months after operation comparing to 1-week postoperation. To quantify the changes of mineral density observed in CT, the standardized linear attenuation coefficient of tissue, measured in Hounsfield units (HU), was evaluated. The HU information is readily available on CT scans without additional costs, radiation, or the use of phantoms. Schreiber et al. described that the HU was so correlated with dual X-ray absorptiometry bone mineral density measurements and mechanical strength that it could be an alternative tool for determining the regional bone mineral density [32]. The results of HU were identical as the observation at coronal view of CT. The HU was gradually increased with time-dependent manner at both groups. The HU at PCL/HA/PBMSCs was significantly higher than PCL/PBMSCs group at all time point due to the high image density property of hydroxyapatite itself. The time-dependent elevation was not significant at PCL/PBMSCs group among each time point. Conversely, the increasing at post-op 2, 4, and 6 mo was significantly higher than post-op 1 wk at PCL/HA/PBMSCs group. These results indicated more new bone formation at the PCL/HA/PBMSCs than PCL/PBMSCs group. Comparing to the HU of normal bone surrounding the defect, we found the HU of PCL/HA/PBMSCs and PCL/PBMSCs at 6 months were around 64% and 22% of that of normal bone, respectively. This means the PCL/HA/PBMSCs could achieve the 64% of mechanical strength of normal bone after 6-month implantation. The histology examination by H&E and Masson's trichrome stain also confirmed more new bone formation within the pore structure of PCL/HA/PBMSCs group. The hardness of the implant tissue-engineered construct after 6 months indicated more mechanical strength at the PCL/HA/PBMSCs than PCL/PBMSCs group. In summary, the animal study revealed more bone formation and stiffness at PCL/HA/PBMSCs group than PCL/PBMSCs group. The possible explanation is the PCL/HA provides a more bone-mimetic environment than PCL. Although the PCL/HA and PCL scaffolds share similar macro- and microstructure by the computer-aided APAD system, the chemical composition and mechanical strength in PCL/HA are closer to bone tissue than PCL. Shih et al. found the matrix stiffness can upregulate the osteogenic gene expression such as type I collagen, osteocalcin, and Runx 2 gene expressions of human MSCs [33]. He et al. demonstrated more bone formation in HA-contained PLG scaffold than the non-HA scaffold because of the increasing scaffold stiffness and bioactive ceramics (HA) [30].

## 5. Conclusion

Based on the outcome of characters of scaffold and* in vitro* osteogenesis study, our study found both scaffolds possess adequate pore size, porosity, and hydrophilicity for cells attachment and proliferation and the PBMSCs expressed upregulation of osteogenic and angiogenic gene in a similar manner on both scaffolds in* in vitro* experiment. The major differences between these two scaffolds were the addition of HA in PCL/HA scaffold, the higher mechanical strength and higher cell proliferation, and VEGF gene expression in PCL/HA scaffold. However, the* in vivo* bone forming efficacy between PBMSCs seeded PCL and PCL/HA scaffold was different from the* in vitro* results. The outcome indicated that the PCL/HA scaffold which had bone-mimetic environment due to the addition of HA obtained the better bone regeneration than PCL scaffold. It is concluded that providing a bone-mimetic scaffold is another crucial factor for BTE except the biocompatible, 3D architecture with high porosity, and interpored connection.

## References

[1] Burg KJ, Porter S, Kellam JF (2000). Biomaterial developments for bone tissue engineering. *Biomaterials*.

[2] Yang S, Leong KF, Du Z, Chua CK (2001). The design of scaffolds for use in tissue engineering. Part I. Traditional factors. *Tissue Engineering*.

[3] Yang S, Leong KF, Du Z, Chua CK (2002). The design of scaffolds for use in tissue engineering. Part II. Rapid prototyping techniques. *Tissue Engineering*.

[4] Yeong WY, Chua CK, Leong KF, Chandrasekaran M (2004). Rapid prototyping in tissue engineering: challenges and potential. *Trends in Biotechnology*.

[5] Sachlos E, Czernuszka JT (2003). Making tissue engineering scaffolds work. Review: the application of solid freeform fabrication technology to the production of tissue engineering scaffolds. *European Cells & Materials*.

[6] Hutmacher DW, Sittinger M, Risbud MV (2004). Scaffold-based tissue engineering: rationale for computer-aided design and solid free-form fabrication systems. *Trends in Biotechnology*.

[7] Jiang CP, Chen YY, Hsieh MF, Lee HM (2013). Solid freeform fabrication and in-vitro response of osteoblast cells of mPEG-PCL-mPEG bone scaffolds. *Biomedical Microdevices*.

[8] Eosoly S, Brabazon D, Lohfeld S, Looney L (2010). Selective laser sintering of hydroxyapatite/poly-*ε*-caprolactone scaffolds. *Acta Biomaterialia*.

[9] Park SA, Lee SH, Kim WD (2011). Fabrication of porous polycaprolactone/hydroxyapatite (PCL/HA) blend scaffolds using a 3D plotting system for bone tissue engineering. *Bioprocess and Biosystems Engineering*.

[10] Shor L, Güçeri S, Wen X, Gandhi M, Sun W (2007). Fabrication of three-dimensional polycaprolactone/hydroxyapatite tissue scaffolds and osteoblast-scaffold interactions in vitro. *Biomaterials*.

[11] Friedenstein AJ, Petrakova KV, Kurolesova AI, Frolova GP (1968). Heterotopic of bone marrow. Analysis of precursor cells for osteogenic and hematopoietic tissues. *Transplantation*.

[12] Pittenger MF, Mackay AM, Beck SC (1999). Multilineage potential of adult human mesenchymal stem cells. *Science*.

[13] Ringe J, Kaps C, Schmitt B (2002). Porcine mesenchymal stem cells: induction of distinct mesenchymal cell lineages. *Cell and Tissue Research*.

[14] Liao HT, Chen CT, Chen CH, Chen JP, Tsai JC (2011). Combination of guided osteogenesis with autologous platelet-rich fibrin glue and mesenchymal stem cell for mandibular reconstruction. *Journal of Trauma*.

[15] Liao HT, Chen CT, Chen JP (2011). Osteogenic differentiation and ectopic bone formation of canine bone marrow-derived mesenchymal stem cells in injectable thermo-responsive polymer hydrogel. *Tissue Engineering C: Methods*.

[16] Lee EJ, Teng SH, Jang TS (2010). Nanostructured poly(*ε*-caprolactone)-silica xerogel fibrous membrane for guided bone regeneration. *Acta Biomaterialia*.

[17] Hulbert SF, Young FA, Mathews RS, Klawitter JJ, Talbert CD, Stelling FH (1970). Potential of ceramic materials as permanently implantable skeletal prostheses. *Journal of Biomedical Materials Research*.

[18] Oh SH, Park IK, Kim JM, Lee JH (2007). In vitro and in vivo characteristics of PCL scaffolds with pore size gradient fabricated by a centrifugation method. *Biomaterials*.

[19] Karageorgiou V, Kaplan D (2005). Porosity of 3D biomaterial scaffolds and osteogenesis. *Biomaterials*.

[20] Roy TD, Simon JL, Ricci JL, Rekow ED, Thompson VP, Parsons JR (2003). Performance of degradable composite bone repair products made via three-dimensional fabrication techniques. *Journal of Biomedical Materials Research A*.

[21] Yeong WY, Sudarmadji N, Yu HY (2010). Porous polycaprolactone scaffold for cardiac tissue engineering fabricated by selective laser sintering. *Acta Biomaterialia*.

[22] Eshraghi S, Das S (2010). Mechanical and microstructural properties of polycaprolactone scaffolds with one-dimensional, two-dimensional, and three-dimensional orthogonally oriented porous architectures produced by selective laser sintering. *Acta Biomaterialia*.

[23] Liao HT, Lee MY, Tsai WW, Wang HC, Lu WC (2013). Osteogenesis of adipose-derived stem cells on polycaprolactone-beta-tricalcium phosphate scaffold fabricated via selective laser sintering and surface coating with collagen type I. *Journal of Tissue Engineering and Regenerative Medicine*.

[24] Sun H, Ye F, Wang J (2008). The upregulation of osteoblast marker genes in mesenchymal stem cells prove the osteoinductivity of hydroxyapatite/tricalcium phosphate biomaterial. *Transplantation Proceedings*.

[25] Harada H, Tagashira S, Fujiwara M (1999). Cbfa1 isoforms exert functional differences in osteoblast differentiation. *Journal of Biological Chemistry*.

[26] Kern B, Shen J, Starbuck M, Karsenty G (2001). Cbfa1 contributes to the osteoblast-specific expression of type I collagen genes. *Journal of Biological Chemistry*.

[27] Lian JB, Stein GS (2003). Runx2/Cbfa1: a multifunctional regulator of bone formation. *Current Pharmaceutical Design*.

[28] Qi H, Aguiar DJ, Williams SM, La Pean A, Pan W, Verfaillie CM (2003). Identification of genes responsible for osteoblast differentiation from human mesodermal progenitor cells. *Proceedings of the National Academy of Sciences of the United States of America*.

[29] Street J, Bao M, DeGuzman L (2002). Vascular endothelial growth factor stimulates bone repair by promoting angiogenesis and bone turnover. *Proceedings of the National Academy of Sciences of the United States of America*.

[30] He J, Genetos DC, Leach JK (2010). Osteogenesis and trophic factor secretion are influenced by the composition of hydroxyapatite/poly(lactide-co-glycolide) composite scaffolds. *Tissue Engineering A*.

[31] Rai B, Lin JL, Lim ZX, Guldberg RE, Hutmacher DW, Cool SM (2010). Differences between in vitro viability and differentiation and in vivo bone-forming efficacy of human mesenchymal stem cells cultured on PCL-TCP scaffolds. *Biomaterials*.

[32] Schreiber JJ, Anderson PA, Rosas HG, Buchholz AL, Au AG (2011). Hounsfield units for assessing bone mineral density and strength: a tool for osteoporosis management. *Journal of Bone and Joint Surgery A*.

[33] Shih YR, Tseng KF, Lai HY, Lin CH, Lee OK (2011). Matrix stiffness regulation of integrin-mediated mechanotransduction during osteogenic differentiation of human mesenchymal stem cells. *Journal of Bone and Mineral Research*.

